# Qualitative risk assessment of homogeneity, stability, and residual concentrations of antimicrobials in medicated feed and drinking water in pig rearing

**DOI:** 10.1186/s12917-022-03555-3

**Published:** 2023-01-13

**Authors:** Despoina Georgaki, Femke Vandael, Helena Cardoso de Carvalho Ferreira, Maria Eleni Filippitzi, Patrick De Backer, Mathias Devreese, Jeroen Dewulf, Siska Croubels

**Affiliations:** 1 Department of Pathobiology, Pharmacology and Zoological Medicine, Laboratory of Pharmacology and Toxicology, Faculty of Veterinary Medicine, Ghent University, Merelbeke, Belgium; 2 Department of Internal Medicine, Reproduction and Population Medicine, Veterinary Epidemiology Unit, Faculty of Veterinary Medicine, Ghent University, Merelbeke, Belgium; 3 Laboratory of Animal Health Economics, Faculty of Veterinary Medicine, Aristotle University of Thessaloniki, Thessaloniki, Greece

**Keywords:** Risk assessment, Oral group treatment, Feed, Drinking water, Homogeneity, Stability, Residual concentrations, Veterinary antimicrobials

## Abstract

**Background:**

Despite the common use of oral group treatment in pig rearing, the magnitude of the factors influencing the homogeneity and stability of antimicrobial drugs in medicated feed and medicated drinking water are largely unknown, as well as the residual concentrations of the drugs after the end of the treatment.

**Results:**

This study presents a qualitative risk assessment to estimate the magnitude of the risks for reduced homogeneity and stability, and increased residual concentrations of antimicrobial drugs in medicated feed and drinking water on the farm. Risk assessment was done using a questionnaire and farm visits (*n* = 52), combined with a second questionnaire, and concentrations of amoxicillin and doxycycline measured in medicated feed and water samples, each collected on 10 farms.

For medicated feed, the duration of storage in the silo did not show to influence the concentration levels in a consistent trend, while the treatment duration had a low to negligible effect. A moderate to high risk was found caused by human error when preparing the medicated feed on the farm. Purchased medicated feed greatly reduces the risk of human error and drugs remain stable during the duration of treatment, while the risk of residual concentrations after the end of the treatment was estimated to be low to moderate. The feed intake variability was identified as a moderate to high risk factor.

For medicated drinking water, the type of dosing pump, age of pre-solution, and human errors during the preparation of the pre-solution present a moderate to high risk on homogeneity and stability. Precipitation of the active substance in the absence of a stirrer in a drinking water tank was shown to be a low to moderate risk factor for residues after treatment. Waterline length had a weak correlation with the concentrations of the antimicrobials, while a moderate to high influence was detected for the water intake by the pigs.

**Conclusions:**

A considerable variation in drug concentration in both medicated feed and medicated drinking water was detected depending on their preparation. Therefore, it is important to know which factors influence the homogeneity and stability, and the residual concentrations after treatment.

**Supplementary Information:**

The online version contains supplementary material available at 10.1186/s12917-022-03555-3.

## Background

Antimicrobial and anthelmintic drugs are frequently used in livestock farming. In the swine industry in particular, antimicrobial drugs consumption is expected to increase the most in comparison to other species, and contributes by 45% to the total increase of antimicrobial use by 2030 [1]. The antimicrobial drugs are commonly administered using medicated feed and medicated drinking water [2, 3]. Oral group treatment is often applied when treating large groups of animals, with a low workload for the farmer. However, treating large groups could result in increased exposure of animals to antimicrobials and consequently increase the selection pressure for antimicrobial resistance [4]. Oral therapy is predominantly used for the administration of medicines to food-producing animals in the European Union. Sales of antimicrobials intended for oral treatment of food-producing animals (using medicated feed or medicated drinking water), and aggregated across 31 European countries, accounted for 86.9% of the total sales in 2020 in Europe [5].

Nevertheless, there is still a lot of uncertainty regarding the likelihood of correct administration of medicated feed or medicated drinking water [2]. For antimicrobial treatment to be efficacious, several parameters play a crucial role: the choice of the medicine, the homogeneity and stability of the drug in feed or water [6, 7], the dose ingested by the individual animal, and drug factors that affect whether or not the pharmacokinetic/pharmacodynamic (PK/PD) breakpoint is reached [8]. Noticeably, high variability in plasma concentrations is observed in individual pigs after the provision of medicated feed [9, 10] and drinking water [11–14]. Several reasons can account for this variability, namely different intake due to social hierarchy and (sub)clinical illness, homogeneity, stability as well as palatability [2, 15–17]. This raises questions regarding the optimal efficacy of treatment and prudent use of antimicrobials [9–11].

The homogeneity and stability of the oral group medication can be influenced in all stages from production or delivery at the farm, up to intake by the animals. Therefore, the procedure followed in the farm needs to be known to identify the critical steps and factors influencing homogeneity and stability. The on-farm process has been described by Vandael et al. [2]. The method of mixing in veterinary medicinal products may influence the homogeneity of the drug. These methods include either mixing by the compound feed manufacturer, or by the farmer using a dosing device on the feeding line in the case of medicated feed, or through a drinking water tank or using an electrical or mechanical dosing pump for medicated drinking water [2]. Also, the duration that the medicated feed/drinking water remains in each stage of the production process can influence the homogeneity and stability due to a.o., temperature, humidity level, pH of the solution, light exposure, segregation [7] and degradation of medicines [18, 19]. For drinking water medication, the solubility of the medication [20], the water quality [21, 22], and the possible use of additives [23] are also important for homogeneity and stability. Vandael et al. (2020) recently demonstrated that antimicrobial drug concentrations in feed were often below the therapeutic concentration range mentioned in the “Summary of Product Characteristics” (SPC), while drinking water concentrations fluctuated between overdosing and underdosing according to the advised target concentration range [24].

The homogeneity and stability of the drug in medicated feed and medicated drinking water must be assured until the moment of intake by the animals. The residual concentrations or in other words the remaining concentrations of the antimicrobial drug in feed or water after the end of treatment, and the homogeneity and stability of medicated feed and medicated drinking water is likely to be influenced by a) the methods used for preparing, transporting, and storing medicated feed and drinking water [25], b) the materials used to construct the feed and drinking water pipelines and their design [26–29], c) the number of treatment days [7], d) the pharmaceutical formulation used [30, 31], and e) the cleaning protocol of the pipelines [2]. Human errors when preparing and administering the medication may also occur if the medication is prepared at the farm instead of bought from a Good Manufacturing Practices (GMP)-certified feed manufacturer [32, 33]. Problems may also occur in every step of the mixing process, which may then result in reduced homogeneity and stability and/or in increased residual concentrations after treatment. Furthermore, the accuracy of the mixing device could also play a role [34, 35].

Previous research also made clear that the composition of delivery systems for medicated feed and drinking water, and the procedures followed by the farmers concerning mixing in the medication, cleaning, and disinfecting, vary widely between farms [2]. Crucial differences were pointed out between the preparation of feed and water medication. First, medicated drinking water is always prepared by the farmer, by using a drinking water reservoir, or an electrical or mechanical dosing pump [2]. On the other hand, medicated feed is, in the majority of cases, bought from the feed manufacturer with a license to produce medicated feed, but it can also be prepared by the farmer using a dosing device on the feeding pipelines [2].

Another relevant risk of oral group medications is the spread of antimicrobial residues [25]. According to European Union regulations, the suggested maximum residual concentration is at 1% of the therapeutic concentration for antimicrobial active substances [36], to avoid the possible rise of resistant bacteria due to exposure to sub-therapeutic concentrations [37]. Filippitzi et al. (2018) designed a risk model attempting to quantify the risk of carry-over of antimicrobial residues in blank feed; feed that was manufactured at the compound feed mill just after mixing in a batch of medicated feed, in a country where medicated feed production corresponds to around 2% of the total feed produced [38]. The model estimated that 5.5% (95% CI = 3.4%; 11.4%) of the total feed produced in a year could be cross-contaminated with different levels of antimicrobials due to practices related to medicated feed. The model also demonstrated that even in cases where medicated feed with antimicrobials would be produced in end-of-line mixers or a fine dosing system on trucks, the risk of cross-contamination would not be negligible. Recently, Vandael et al. (2020) demonstrated that drug residual concentrations measured 2 days after the end of therapy with both feed and water medication rarely exceeded 1% of the lowest therapeutic concentration in their study [24]. Taking the findings from Vandael et al. (2020) as a starting point, this present study focuses on the risk of a given farm administering medicated feed or drinking water for a reduced homogeneity and stability, and for increased residual concentrations after the end of treatment, using amoxicillin (AMO) and doxycycline (DOX). It was opted to use a qualitative risk assessment rather than a quantitative one: one for medicated feed and one for medicated drinking water. Characterizing risks can lead to more informed measures to limit the magnitude of such risks, eventually leading to more targeted approaches and more efficient group medication strategies.

## Results

**Fig. 1:**
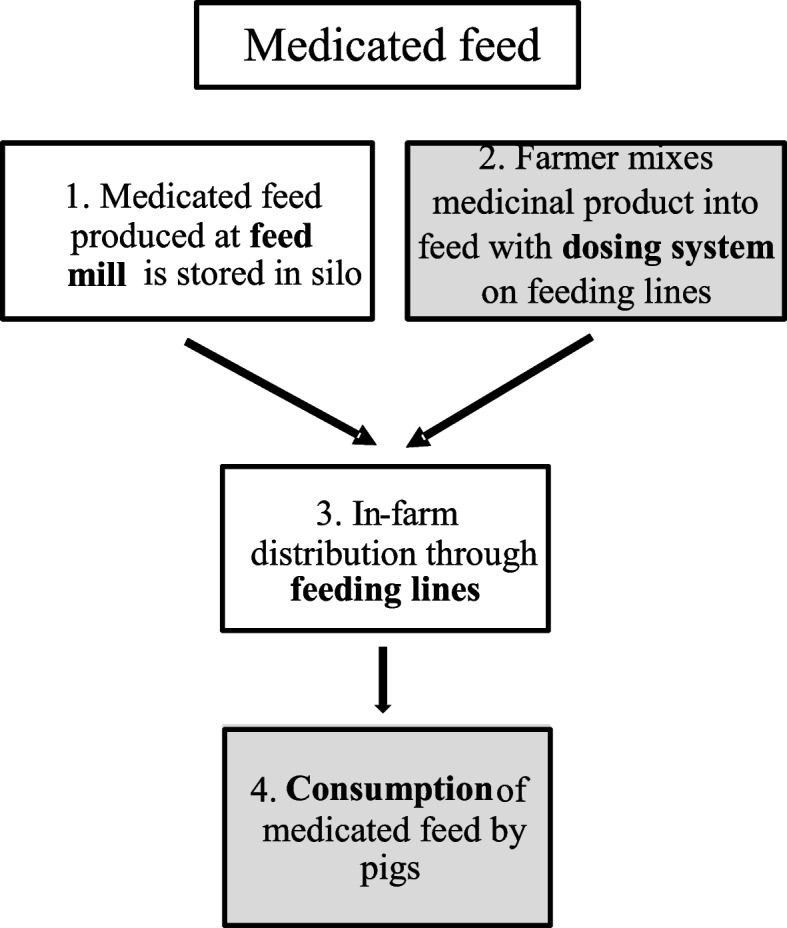
Risk assessment scheme representing the likelihood of medicated feed having poor homogeneity and poor stability. Starting from delivery at the farm or production by the farmer and ending with consumption by the pigs. The assessment is based on 4 modules. Modules where a moderate to high risk of reduced stability and/or homogeneity occurs, are colored grey

The results of the qualitative risk assessment are presented for medicated feed and medicated water. In Fig. 1, the risk assessment scheme for reduced homogeneity and stability of medicated feed is shown.

### Medicated feed

- Module 1: medicated feed produced at the compound feed mill is stored in a silo

A possible risk factor for reduced homogeneity is associated with the installed treatment and is identified as the number of treatment days. It is hypothesized that longer treatment regimens imply that medicated feed will be stored in the silo for a longer period, thereby increasing the risk of segregation. In Table 1, the concentrations of AMO and DOX in medicated pig feed are presented, combined with the method of preparing the medicated feed and the number of treatment days (bottom row). Farms 3 to 10 purchased their medicated feed from the feed mill. The concentration levels did not show a consistent trend throughout the different time-points during the treatment (beginning, middle, and end); with some results falling below the therapeutic concentration range based on the SPC or leaflet and at later sampling points returning to acceptable levels, concluding as weak correlation between frequency of underdosing observations and number of treatment days. Furthermore, the correlation between longer treatment duration [number of treatment days] and reduction in homogeneity was shown to have a weak correlation (r = − 0.29), and therefore the risk was characterized as low to negligible. However, farm 7 was found to have significant residual concentrations even at 2 days after the end of the treatment period since more feed was provided to the silo than it was needed, concluded to an undesired and inevitable longer treatment. On all other farms, the residual concentrations present at 2 days after the end of treatment were negligible.

- Module 2: farmer mixes a veterinary medicinal product into the feed using a dosing system on the feeding lines

**Table 1:**
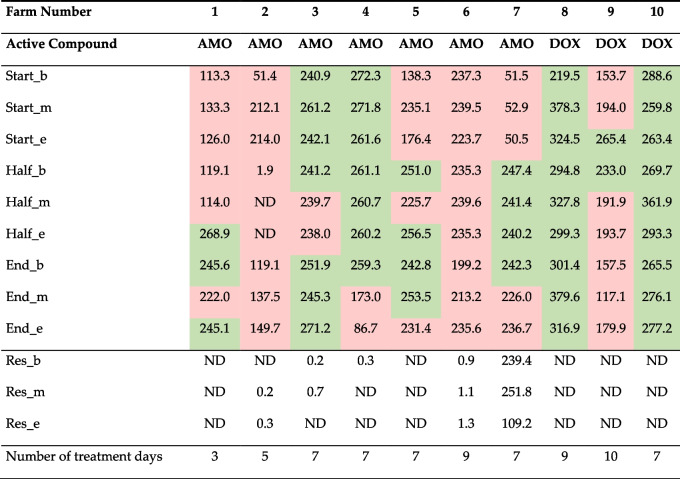
Mean concentrations of amoxicillin (AMO) and doxycycline (DOX) in medicated feed samples

Mean concentrations of duplicate measurements are shown in μg/g, and were obtained in samples collected at the start of treatment and sampled at the beginning (start_b), middle (start_m,) and end (start_e) of the feed line; as well as sampled halfway through the treatment (again at the beginning (half_b), middle (half_m), and end (half_e) of the feed line), on the last day of treatment (at the beginning (end_b), middle (end_m), and end (end_e) of the feed line), and 2 days after the end of the treatment to measure the residual concentrations at the beginning (res_b), middle (res_m), and end (res_e) of the feed line. Values shown in green were within the therapeutic concentration range for that active substance (240–550 μg/g AMO; 200–550 μg/g DOX), after taking into account the measurement uncertainty of the analytical method (− 20 to + 10%); values in red were below the therapeutic concentration range. Values lower than the detection limit were presented as ND (not detected). Medicated feed was produced at the feed mill with an end-of-line mixer (*n* = 8), or by the farmer using a dosing system on the feed line (*n* = 2, farm number 1 and 2). Adapted from Vandael et al., 2020 [24]

As an alternative to purchasing medicated feed, the farmer can prepare medicated feed using a dosing device on the feeding lines. Possible considered risk factors here were associated with the preparation procedure and were characterized by a) the occurrence of human errors when weighing medication or when the device is not switched on, b) and the calibration of the dosing device itself. A dosing device was used in farms 1 and 2 (Table 1). Data from the questionnaire revealed the farmer in the second farm forgot to switch on the dosing device halfway through the treatment period and this instance had detrimental effects on the concentrations of AMO measured (levels below the limit of detection, Table 1) [24]. Even though this could be considered a punctual episode, human errors can have a substantial impact on the concentrations of drugs and were therefore classified as being a ‘high to a very high’ risk factor, as they are likely to result in a reduced homogeneity.

- Module 3: in-farm distribution of medicated feed through the feeding lines

After the medicated feed leaves the silo or the dosing device, it is transported to the feeding troughs through the feeding pipelines. In theory, segregation, and consequently reduced homogeneity, can take place over the length of the pipelines. This was hypothesized to eventually result in a reduced concentration at the end of the feeding troughs. However, no obvious trend was observed between the concentration measured at the beginning, middle, and end of the feeding line, being in general quite stable with little variation during all stages of the treatment across types of medication (Table 1). The correlation between the mean drug concentration and the length of the feeding line was r = 0.11 for the cases of AMO and r = 0.25 for DOX. Therefore, the risk of a reduced homogeneity of medicated feed caused by the length of the feeding pipelines was classified as ‘low to negligible’.

- Module 4: consumption of medicated feed by the pigs

It was not possible to study the consumption of the medicated feed by the (individual) pigs in the current study. However, other studies [9, 10, 15] show a large variability in feed consumption between pigs housed in the same pen and stable. Given this large variability, the risk is estimated to be ‘moderate to high’ based on available literature.

### Medicated drinking water

**Fig. 2:**
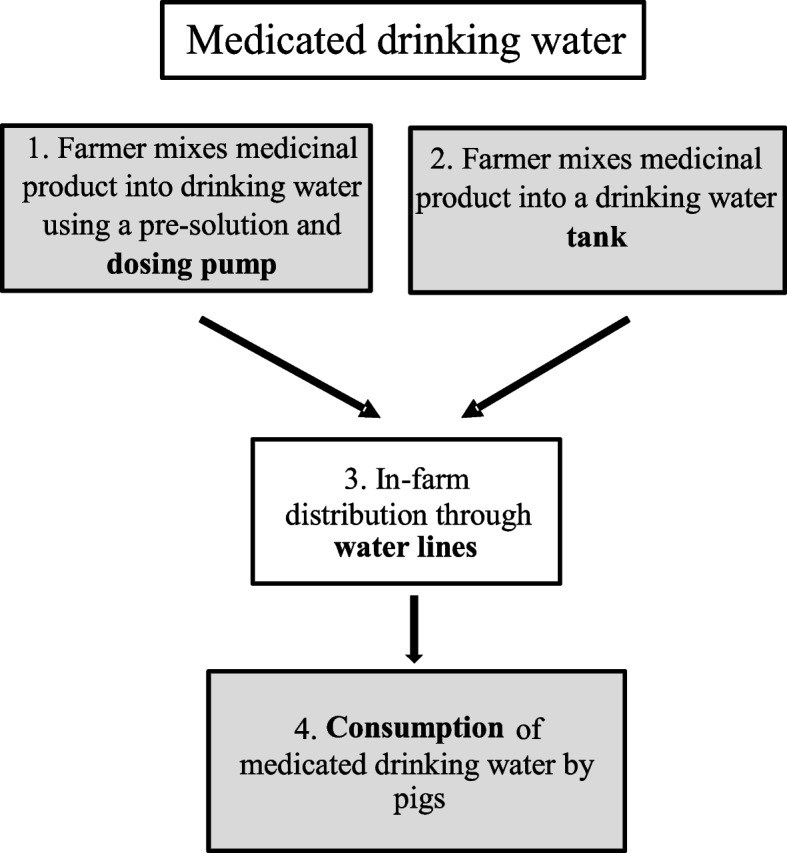
Risk assessment scheme representing the likelihood of medicated water having poor homogeneity and poor stability. Starting from production by the farmer and ending with consumption by the pigs. The assessment is based on 4 modules. Modules where a moderate to high risk of reduced stability and/or homogeneity occurs, are colored grey

In Fig. 2, the risk assessment scheme for reduced homogeneity and stability of drinking water medication is shown. Regardless of using a dosing pump or a drinking water reservoir, the drinking water quality is important. When using drinking water, additives were used at 5 farms, consisting of sodium bicarbonate or a flavoring agent.

- Module 1: farmer mixes a veterinary medicinal product into drinking water using a pre-solution and an electrical or mechanical dosing pump

Medicated drinking water was mixed in using a mechanical (*n* = 6) or electrical (*n* = 2) dosing pump. According to the results shown in Table 2, over- and underdosing of drinking water medication was observed quite frequently. There was a moderate to high effect of the type of dosing pump used (mechanical or electrical) on the homogeneity of the antimicrobial drug (r = − 0.65 for AMO and r = − 0.57 for DOX), indicating that a mechanical pump concludes to less variation on the homogeneity of both drugs. Possible risk factors when using a dosing pump can be the absence of calibration or poor maintenance of the pump, which was observed in the vast majority (79%) of the farms using dosing pumps [2]. The accuracy of the dosing pump is unknown, similar to what was observed in the feed medication risk assessment for the cases that the medicated feed was not prepared by the manufacturer. The increased age of the pre-solution has been shown to conclude to underdosing (farm 6 with a pre-solution of 60 hours old for AMO), highlighting the importance of the age factor on the stability of the drug in the solution. Another risk factor considered to be important was the possibility of human error in weighing the medication. In the study from Vandael et al. (2019), 6.4% of the farmers visually estimated the amount of veterinary medicinal product to be mixed in, 21.3% used a measuring cup, and 75.0% used a measuring scale [2]. Mixing an increased or decreased amount of veterinary medicinal product into drinking water using a pre-solution and an electrical or mechanical dosing pump, has been identified as a ‘moderate to high’ risk factor.

- Module 2: farmer mixes a veterinary medicinal product into a drinking water tank

**Table 2:**
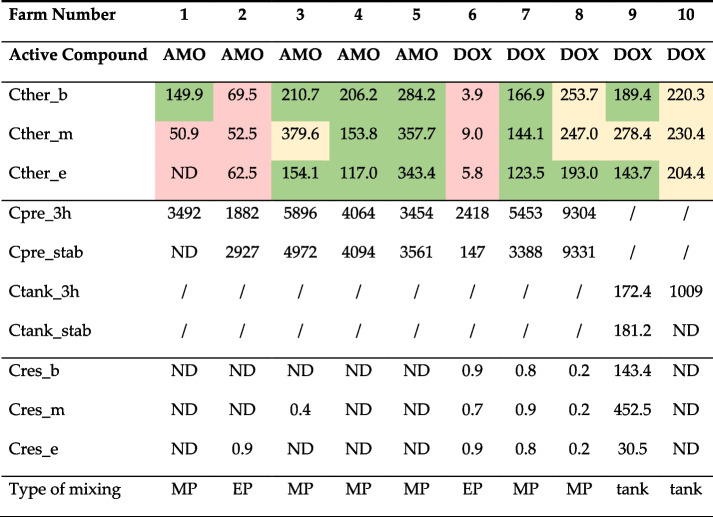
Mean concentrations of amoxicillin (AMO) and doxycycline (DOX) in medicated drinking water samples (duplicate measurements), in μg/ml

The concentration of samples taken during treatment was taken 3 hours after the start of the treatment and this at the start (Cther_b), at the middle (Cther_m), and end (Cther_e) of the drinking water pipeline. The pre-solution (in case a dosing pump was used) and the drinking water tank were sampled after 3 h (Cpre_3h/Ctank_3h) and just before the pre-solution/tank was finished or just before a new batch of pre-solution/tank was prepared to determine its stability (Cpre_stab/Ctank_stab). Samples were also taken 2 days after the end of the treatment to measure the residual concentrations at the beginning (Cres_b), middle (Cres_m), and end (Cres_e) of the drinking water pipeline. Values shown in green were within the therapeutic concentration range for that active substance, with the uncertainty of the analytical methods taken into account (−20 to + 10%; 106–368 μg/ml AMO; 82–199 μg/ml DOX). Values in red were below the therapeutic concentration range, values higher than the therapeutic concentration range are shown in yellow. Values lower than the detection limit are presented as ND (not detected). Medicated drinking water was prepared using a mechanical (MP) or electrical (EP) dosing pump, or a drinking water tank (tank). Adapted from Vandael et al., 2020 [24]

Only two of the sampled farms, numbers 9 and 10, used a drinking water tank. Farm 9 showed high residual concentrations at 2 days after the end of the treatment with DOX. Here, the medication was added to a drinking water tank without a stirrer, probably resulting in the accumulation of the medication at the bottom of the drinking water tank. This increases the risk of residual concentrations and spread downstream, however on all other farms, the residual concentrations present at 2 days after the end of treatment were negligible. Therefore, the risk is classified as ‘low to moderate’.

- Module 3: in-farm distribution through water lines

Medicated drinking water is transported to the drinking nipples via water pipes. Analogous to the risk assessment for medicated feed, there was no clear effect of the length of the water pipes on the measured concentration at the beginning, middle, and end of the water pipes, and concentrations decreased gradually (particularly for DOX). Therefore, the risk of this factor for a reduced homogeneity was estimated to be ‘low to moderate’, with r = − 0.22 for AMO and r = − 0.40 for DOX. Another possible risk factor was the material from which the pipelines were made. However, nine out of 10 samples were taken at nipples supplied by polyvinyl chloride (PVC) water lines, and only one by stainless steel water lines, so no comparison could be made, and the risk was characterized as unknown.

- Module 4: consumption of medicated drinking water by pigs

The uptake of medicated water by the (individual) pigs was unknown. However, research by other groups [39–42] shows large individual variability in water consumption, as well as an influence of the environment [43], time of day [44], age [45], health status [15, 46], and social hierarchy [40]. Given the large, expected effect of these factors on the medicated water uptake, the risk is classified as ‘moderate to high’ in this module.

## Discussion

The majority of medicinal products for pigs are administered using oral group treatment by medicated feed and drinking water. For the oral group treatment to be effective, three pillars are important according to Soraci et al. (2014) [40]: a) the concentration of the active compound in the medicated feed in the feeding troughs, or medicated drinking water at the drinking nipples, which needs to be correct; b) the amount of feed or drinking water that is consumed by the pigs, which needs to be adequate, and c) the variability in pharmacokinetic parameters such as oral bioavailability, volume of distribution and clearance between pigs needs to be known. Yet, the factors influencing the homogeneity, stability, and cross-contamination of medicated feed and drinking water are not thoroughly investigated at the farm level. Previous research characterized these factors, by using a questionnaire and by analysing samples of medicated feed and drinking water on 20 pig farms [24]. Samples containing AMO and DOX were taken at different sampling sites at the farm, from the pre-solution to the feeding troughs and drinking nipples.

In summary, the qualitative risk assessment for medicated feed provides the following classification of risk factors: 1) a low to negligible risk is attributed to the duration of treatment (in days); 2) a high to very high risk is attributed to the use of a dosing device on the feeding line (as result of human error) and unknown risk to the accuracy of the dosing device; 3) a low to negligible risk for the length of the feeding lines to influence the homogeneity of the drugs; and 4) a moderate to high risk is attributed to the variability of feed consumption by the pigs. The risk of residual concentrations being present after the end of treatment is estimated to be low to moderate, based on the results from the study by Vandael et al. (2020) and Filippitzi et al. (2018) [24, 38].

In addition, the qualitative risk assessment for medicated drinking water gave the following classification of risk factors: 1) a moderate to high risk of human error in weighing the veterinary medicinal product, the age of the pre-solution, and the type of the dosing pump, even though the accuracy of the dosing pump was an unknown risk factor; 2) a low to moderate risk of precipitation of active substance in the absence of a stirrer in a drinking water tank; 3) a low to moderate risk concerning the influence of the length of the water pipes on the homogeneity and stability of the drugs; and 4) a moderate to high risk is attributed to the variability in medicated water intake by the pigs.

A risk model has been previously designed to predict the risk of carry-over of antimicrobial residues in blank feed batches, when feed is manufactured at the compound feed mill after mixing in a batch of medicated feed [38]. The values used in this model were worst-case estimated values, due to the lack of information about the concentrations measured at the farm level. In contrast, Vandael et al. (2020) obtained samples to measure residual concentrations at 2 days after the end of the treatment, instead of immediately after the production of medicated feed, resulting in a low to moderate risk of residues [24]. Therefore, the focus in the present study was to perform a risk assessment predicting a reduced homogeneity and stability, instead of focusing on the carry-over of residues. Unfortunately, the data collected were unsuitable for the design of quantitative models because the power was too low to infer statistically significant associations. Therefore, the risk assessment presented in this study is qualitative. The use of models always has limitations, and they cannot provide an exact identification of the present or future risk [47].

Results showed that medicated feed purchased and stored in the silo remained homogeneously mixed during the duration of the treatment (3 to 10 days). However, every day of treatment results in an exposure of the animals to antimicrobial drugs, and therefore, the duration of treatment should be kept as limited as possible [48].

One of the goals of this study was to assess the effect on the homogeneity of on-farm preparation of medicated feed compared with medicated feed prepared by the feed mill. Given that medicated feed purchased from the feed mill is produced following GMP guidelines, human errors are far less likely than when medicated feed is prepared by the farmer using a dosing device on the feeding lines. However, a dosing device was only used in 2 out of 10 farms applying medicated feed, which limits the conclusions to be drawn from this data.

Another factor to consider is the variability in consumption of medicated feed, which is likely to be an important risk factor in reaching sufficiently high plasma concentrations. Such data were not available in this study, but other studies have investigated the variability of consumption of medicated feed and indeed demonstrated variable intake by the animals, e.g. due to social hierarchical status [9–11, 15, 39]. Future studies focusing on individual monitoring of feed consumption could lead to more accurate dosing of medicinal products in the medicated feed, where the amount of active product is adjusted to the feed consumption by the animals [49].

When using medicated drinking water, the drinking water quality is of the utmost importance and regular annual testing is advised. Since the chemical drinking water quality was sufficient for livestock use in all farms, the effect of the drinking water quality could not be studied here. The pharmaceutical formulation of the veterinary medicinal products is also a pivotal influencing factor on the stability and homogeneity (e.g. precipitation, solubility issues) of the drinking water medication [31, 50].

Additional requirements for therapy with medicated drinking water are the use of medicinal products with good solubility, good drinking water quality, and preferably a separate pipeline system for medicated and non-medicated water. Furthermore, the use of additives should also be taken into account as these can change the drinking water pH for instance, and affect solubility [2, 51]. However, to test the influence of water quality, formulation, and additives, a study in a controlled environment is necessary, rather than a field study.

Two main types of providing medicated drinking water can be distinguished, namely using a dosing pump, or a drinking water reservoir. The latter is associated with more problems regarding poor hygiene since it is more difficult to clean, and a large volume of still water may promote the growth of bacteria and algae. Cases of high residual concentrations (Cres > 30 μg/ml) were only seen on one farm, where a drinking water reservoir without a stirrer was used. This highlights the importance of a stirring device to keep the drug homogeneously mixed. Dosing pumps are either electrical (more expensive, but more accurate) or mechanical (pre-solution is mixed in pulses, deemed less accurate, but also less expensive), although this study indicated a mechanical pump results in less variation on the homogeneity of both AMO and DOX. However, the sample size in this study is too small and other reasons of variability, such as human error when preparing the medicated drinking water, are more probable to cause a distinctive difference in accuracy than the type of dosing pump used. However, many currently registered medicinal products are not suitable for use in a dosing pump as the solubility is generally too low.

Drinking water medication is always prepared at the farm, making it prone to human error. In human medicine, the occurrence of human error when preparing medication is a known problem [32, 33], but such research in veterinary medicine seems to be scarce. Using a scale when preparing the medication is essential for accurate dosing, but a previous study [2] showed some farmers estimate the required amount of medicinal products by sight (6.4%), or use a measuring cup (21.3%). The package leaflet states after how many hours a pre-solution must be refreshed. This period (at least every 24 hours, or 12 hours in the case of AMO) must be respected. Farm 6 had a pre-solution of AMO which was 60 hours old, resulting in serious underdosing due to stability issues of this drug in solution [52].

The material of the pipelines can be a risk factor for a reduced stability of the drug in drinking water. Reports show that galvanized steel is much less inert than PVC or stainless steel [28]. In the sampled farms, however, PVC was ubiquitous (*n* = 9), which made it impossible to evaluate the influence of the pipeline material on the measured concentration.

The variation in water intake is likely to be a major risk factor in under- or overdosing and therefore was taken into account in the risk assessment. Water consumption is known to have large individual variability, and it varies during the time of day [53], the ambient temperature [54], group hierarchy [40], and disease state [15]. Although critical for accurate dosing, water consumption is usually roughly estimated and rarely measured by the farmer [2, 35, 55]. Since the number of animals to be treated tends to vary, the accuracy and consistency when estimating water consumption are sure to be under- or overestimated. Even if a water meter is used to monitor consumption, there still is the issue of water wastage by the pigs [39, 56].

## Conclusion

To conclude, the results show a considerable variation in drug concentration in both medicated drinking water and medicated feed if prepared by the farmer, in contrast to medicated feed purchased from the feed mill. In the latter, the concentration remains more consistent during treatment and between different farms. Both medicated feed and medicated drinking water were frequently underdosed, and drinking water sometimes overdosed. Only on 2 out of 10 farms using medicated feed, and in 3 out of 10 farms using medicated drinking water, the therapeutic concentration range was met [24]. Therefore, it is important to know where the homogeneity and stability of the medicated feed and drinking water diminish during the preparation and transport of medicated feed and drinking water at the farm.

The recommendations for the correct use of medicated feed and drinking water include the training of veterinarians and pig farmers, correct preparation and thorough mixing of medicated drinking water, respecting the posology and advice from the package leaflet, monitoring the drinking water quality, and changing the pre-solution sufficiently frequent. The possibility of human error needs to be reduced. More research is needed for further elaboration of the risk assessment, by taking more samples of medicated feed and medicated drinking water on a higher number of farms, of more active compounds, preferably in combination with the determination of plasma concentrations.

## Methods

The qualitative risk assessments for medicated feed and medicated drinking water aim at examining the risk of a reduced homogeneity and stability, and increased residue levels. Both were performed using the methodology described by the WHO as a guideline. After summarizing the collected data from Vandael et al. (2020) [2, 24], the following steps were taken: a) hazard identification, to identify risk factors causing a reduced homogeneity/stability, and/or an increase in residual concentrations after the end of treatment; b) hazard characterization, to evaluate the nature of these risk factors, and to estimate the magnitude of their importance; c) exposure assessment, to describe the pathway(s) how the medicated feed and drinking water can present an increased risk of reduced homogeneity/stability and increase in residual concentrations, and to estimate the likelihood of this exposure to occur; and finally d) risk characterization, which integrates the results from both the hazard characterization and the exposure assessment.

After these steps were taken, it was possible to estimate the probability of occurrence and severity of the risk factors. Risk assessments were performed using data from a questionnaire and farm visits (*n* = 52) described elsewhere [2], and from a second questionnaire and concentrations of AMO and DOX measured in samples of medicated feed collected on 10 pig farms (*n* = 7 for AMO, *n* = 3 for DOX) and medicated drinking water from 10 other farms (n = 5 for AMO, n = 5 for DOX) [24].

### Questionnaire data

Following the process from the preparation of medicated feed and water to the intake by the pigs, questionnaire data from 52 farms [2] were used for identifying and characterizing the risk factors that could influence homogeneity/stability and residual concentrations after the end of treatment. Specifically, the questions were focusing on: a) the treatment installed (formulation, number of treatment days), b) the pigs being treated (production category, group size (i.e. per pen, per compartment, or stable), and clinical symptoms (if present)), c) the feeding and drinking water system including details on the type of feed/water used, pipeline materials, water purification, and finally d) the preparation of the medicated feed or medicated drinking water.

The medicated feed was either purchased or mixed in by using a dosing system on the feeding line for feed medication; medicated drinking water was either mixed in by a dosing pump (electrical or mechanical) or using a drinking water reservoir. Additional information was recorded on the use of additives in the case of water medication. The distance from the silo to the first feeding trough was noted in the case of medicated feed.

The pig farmers were asked to sign a form of consent to participate in the study and fill questionnaires concerning group treatment with medicated feed and drinking water. An English version of the questionnaire is added as Additional file 1: supplementary file.

### Data on antimicrobial drug concentrations in medicated feed and medication drinking water

Following the steps of risk identification and characterization, the antimicrobial drug concentrations measured in medicated feed and drinking water were used to perform both assessments and are based on the results described by Vandael et al. (2020) [24]. In short, samples in the referred study were collected on pig farms when treatment with AMO or DOX was prescribed via feed or drinking water medication. Feed samples were taken on the first, middle, and last day of the treatment period, and 2 days after the end of the treatment to study residual concentrations or cross-contamination. For each time point, samples were taken at the beginning, middle, and end of the feeding line (Table 1).

On the other hand, medicated drinking water samples were taken 3 hours after the preparation of a fresh batch of drinking water medication, and 2 days after the end of the treatment. For each time point, medicated water samples were also taken at the beginning, middle, and end of the waterline, at the drinking nipples (Table 2). The antimicrobials were quantified in all samples using validated liquid chromatography coupled to tandem mass spectrometry (LC-MS/MS) methods. Further information can be found in Vandael et al. (2020) [24].

### Data used for risk assessment

Details on the construction of the systems to store and prepare medicated feed or medicated drinking water have been previously described by Vandael et al. (2019) [2]. This information was used to perform risk assessments using a modular approach, with each module representing a process in the chain starting with the production of the medicated feed or drinking water; and ending in consumption of both types of medication by the pigs.

Each approach consists of four modules. For medicated feed (Fig. 1), these were: 1) medicated feed produced at the feed mill and stored in a silo; 2) medicated feed prepared by the farmer by mixing a veterinary medicinal product into feed by using a dosing system on the feeding lines; 3) in-farm distribution through feeding lines; 4) consumption of medicated feed by the pigs. For medicated drinking water (Fig. 2), the four modules were: 1) medicated drinking water prepared by the farmer using a pre-solution and electrical or mechanical dosing pump; 2) medicated drinking water prepared by the farmer using a drinking water tank; 3) in-farm distribution through water lines; 4) consumption of medicated drinking water by the pigs.

For both assessments, module 4 is not directly related to the risk for a reduced homogeneity and stability, or increased residual concentrations; but it was taken into account as it is also a crucial factor leading to an efficient antimicrobial therapy.

The results of both assessments are discussed per module, successively listing the hazard identification, hazard characterization, and exposure assessment. These assessments take into account homogeneity and stability together. The risk of accumulation of residual concentrations is considered separately.

### Ranking of risk factors and risk characterization

As last step of the risk assessment, questionnaire data from 20 farms [24] were compared with available literature information and then combined with the concentrations measured in the samples of medicated feed and drinking water to make correlations between the two, using Pearson Correlation Coefficient. The risk was estimated if a) an association could be found between the results from the questionnaire and the measured concentration, or b) a factor influencing the homogeneity, stability and residual concentrations was identified. A rank was assigned to each identified risk pathway, depending on the correlation results between the different concentrations of the drugs and parameters considered as factors affecting homogeneity, stability, and residual concentrations. According to this ranking, the risk was identified as ‘negligible to low’ when the absolute correlation value fluctuates between 0 and 0.3, ‘moderate’ between 0.3 and 0.7, ‘high to very high’ between 0.7 and 1.0, and ‘unknown’ when the data were not available or with sample size less than two [57]. This ranking of risk factors was based on the tendencies observed from the questionnaire combined with results from previously published literature on risk factors affecting feed and water medication, such as the preparation, storage and distribution methods of the medicated feed and drinking water, construction material of the pipelines, the duration of the treatment, and the individual intake of medicated feed and drinking water [2, 24, 38, 41, 43, 44].

## Supplementary Information

**Additional file 1.**

## Data Availability

The datasets used and analysed during the current study are available from the corresponding author upon request.

## Abbreviations

AMO: amoxicillin
CI: confidence interval
DOX: doxycycline
GMP: Good Manufacturing Practices
LC-MS/MS: liquid chromatography coupled to tandem mass spectrometry
PK/PD: pharmacokinetic/pharmacodynamic
PVC: polyvinyl chloride
SPC: Summary of Product Characteristics
