# Supplementary material for: Qualitative risk assessment of homogeneity, stability, and residual concentrations of antimicrobials in medicated feed and drinking water in pig rearing
Source: BMC Vet Res. 2023 Jan 13;19:9. doi: 10.1186/s12917-022-03555-3 (PMC9837973; doi:10.1186/s12917-022-03555-3)
Supplement: Supplementary file 1 — Additional file 1. [file 12917_2022_3555_MOESM1_ESM.pdf]

FACULTY OF VETERINARY MEDICINE
accredited by EAEVE

GHENT UNIVERSITY

LABORATORY OF PHARMACOLOGY AND TOXICOLOGY

Survey on drinking water and feed systems in pig farming, used for group medication

GROUPMEDIPIG

Tick what fits. When the survey mentions sows, it means sows in group housing.

General farm information

1. Number of sows currently present: ……………………………………………
2. Number of weaned piglets currently present: ……………………………………………
3. Number of fattening pigs currently present: ……………………………………………

Question	Drinking water	Feed
**Structure of the drinking water/feed system**		
4. What kind of water/feed do you use for your sows? (Several answers are possible)	☐ Main water/city water<br>☐ Spring water from well/deep well/pulse well<br>☐ What is the depth of the well? .... ☐ Drainage water<br>☐ Surface water<br>☐ Rain water	☐ Flour<br>☐ Granules/pellets<br>☐ Wet/dry feed (pigs mix water in their dry feed themselves)<br>☐ Liquid feed (wet feed)<br>☐ Other, i.e. ....

Question	Drinking water	Feed
5. What kind of water/feed do you use for your piglets? (Several answers are possible)	☐ Main water/city water<br>☐ Spring water from well/deep well/pulse well<br>☐ What is the depth of the well? .... ☐ Drainage water<br>☐ Surface water<br>☐ Rain water	☐ Flour<br>☐ Granules/pellets<br>☐ Wet/dry feed (pigs mix water in their dry feed by themselves)<br>☐ Liquid feed (wet feed)<br>☐ Other, i.e. ....
6. What kind of water/feed do you use for your fattening pigs? (Several answers are possible)	☐ Main water/city water<br>☐ Spring water from well/deep well/pulse well<br>☐ What is the depth of the well? .... ☐ Drainage water<br>☐ Surface water<br>☐ Rain water	☐ Flour<br>☐ Granules/pellets<br>☐ Wet/dry feed (pigs mix water in their dry feed by themselves)<br>☐ Liquid feed (wet feed)<br>☐ Other, i.e. ....
7. How old is the oldest part of the water/feed supply approximately?	………………………………………………………………………………………… .	………………………………………………………………………………………………

8. How old is the newest part of the water/feed supply approximately?	……………………………………………………<br>……………………………… .	………………………………………………<br>………………………………………………
9. Do you have a water meter?	☐ Yes<br>☐ No	
10. What material are the main pipes made of (several answers are possible)	☐ Iron<br>☐ Galvanised iron<br>☐ PVC<br>☐ Stainless steel<br>☐ Other, namely<br>..	☐ Iron<br>☐ Galvanised iron<br>☐ PVC<br>☐ Stainless steel<br>☐ Steel coated with enamel<br>☐ Other, namely<br>..
**Question**	**Drinking water**	**Feed**
11. What material are the pipes in the sows compartment made of?	☐ Iron<br>☐ Galvanised iron<br>☐ PVC<br>☐ Stainless steel<br>☐ Other, namely<br>..	☐ Iron<br>☐ Galvanised iron<br>☐ PVC<br>☐ Stainless steel<br>☐ Steel coated with enamel<br>☐ Other, namely<br>..
12. What material are the pipes in the piglets compartment made of?	☐ Iron<br>☐ Galvanised iron<br>☐ PVC<br>☐ Stainless steel<br>☐ Other, namely<br>..	☐ Iron<br>☐ Galvanised iron<br>☐ PVC<br>☐ Stainless steel<br>☐ Steel coated with enamel<br>☐ Other, namely<br>..

Question	Drinking water	Feed
13. What material are the pipes in the fattening pigs compartment made of?	☐ Iron<br>☐ Galvanised iron<br>☐ PVC<br>☐ Stainless steel<br>☐ Other, namely	☐ Iron<br>☐ Galvanised iron<br>☐ PVC<br>☐ Stainless steel<br>☐ Steel coated with enamel<br>☐ Other, namely ....
**Water purification, water quality and acidification**		
14. If you purify the drinking water, how do you do it?	……………………………………………………<br>……………………………………………………<br>……………………………………………………<br>……………………………………………………<br>……………………………………………………<br>……………………………………………………<br>………………	/
**Question**	**Drinking water**	**Feed**
15. Do you have a water sample examined?	☐ Never<br>☐ Yes→ how often? ……………………………………………<br>……………………………………………<br>……………………………………………<br>……………………………………………<br>…………………………………	/
16. If you have a water sample examined, where do you take the samples (several answers possible)?	☐ At the source (the water entering the establishment)<br>☐ At the water tank<br>☐ At the drinking troughs/drinking nipples	/
17. Do you acidify the sows' water? Which products do you use?	……………………………………………………<br>……………………………………………………<br>……<br>……………………………………………………	/

	………………………………………………………… …………………………………………………	

Question	Drinking water	Feed
18. Do you acidify the piglets' water? Which products do you use?	…………………………………………………………… ……………………………………………………………… …… ………………………………………………………… ………………………………………………………… ………………………………………………………… ………………………………………………………… … ….	/
19. Do you acidify the water for the fattening pigs? Which products do you use?	…………………………………………………………… ……………………………………………………………… …… ………………………………………………………… ………………………………………………………… ………………………………………………………… ………………………………………………………… … ….	/

Questions	Drinking water	Feed
**Cleaning and disinfection**		
20. How often is the following cleaned and/or disinfected? (For example: the pipes are cleaned once a week)	☐ Water pipes .... ☐ Water tank .... ☐ Drinking troughs/nipples	☐ Silo .... ☐ Feed mixing tank (if any) .... ☐ Feed troughs ....
21. With which products are the above parts cleaned and disinfected? (Several answers are possible)	☐ Hydrogen peroxide ☐ Mixture of organic acids ☐ Chlorine dioxide	☐ Hydrogen peroxide ☐ Mixture of organic acids ☐ Chlorine dioxide

	☐ Other, namely	☐ Other, namely ....
**Question**	**Drinking water**	**Feed**
**Group treatment via drinking water or feed**		
22. Which compartments can receive medication via drinking water or feed?	☐ Sows<br>☐ Piglets<br>☐ Fattening pigs<br>☐ I never use drinking water medication	☐ Sows<br>☐ Piglets<br>☐ Fattening pigs<br>☐ I never use feed medication
23. Are combinations of medicines sometimes added to the drinking water/feed?	☐ Yes namely,<br>……………………………………………<br>……………………………………………<br>……………………………………………<br>……………………………………………<br>……………………………………………<br>…………………………………………….<br>☐ No, never	☐ Yes namely,<br>……………………………………………<br>……………………………………………<br>……………………………………………<br>……………………………………………<br>……………………………………………<br>……………………………………………<br>☐ No, never
24. Which groups of sows can be given separate (independent) drinking water/feeding medication?	☐ Per house<br>☐ Per compartment<br>☐ Per stable	☐ Per house<br>☐ Per compartment<br>☐ Per stable
**Question**	**Drinking water**	**Feed**
25. Which groups of piglets can be given separate (independent of each other) drinking water/feed medication?	☐ Per house<br>☐ Per compartment<br>☐ Per stable	☐ Per house<br>☐ Per compartment<br>☐ Per stable
26. Which groups of fattening pigs can be given separate (independent) drinking water/feed medication?	☐ Per house<br>☐ Per compartment<br>☐ Per stable	☐ Per house<br>☐ Per compartment<br>☐ Per stable
27. Is there a separate water/feed line for medicated water/feed and for water/feed to which no medication has been added?	☐ Yes<br>☐ No	☐ Yes<br>☐ No
**Preparation of feed and water medication**		
28. Are the pipes cleaned before starting the drinking water/feed treatment?	☐ Always<br>☐ Sometimes	☐ Always<br>☐ Sometimes

	☐ Never	☐ Never
29. Are the pipes cleaned after adding drinking water/feeding medication?	☐ Always<br>☐ Sometimes<br>☐ Never	☐ Always<br>☐ Sometimes<br>☐ Never
**Question**	**Drinking Water**	**Feed**
30. By what means do you take medication?	☐ Via a dosing pump<br>☐ Electrical doser (Digi Doser, Aquados, MSeasydoser,...)<br>☐ Mechanical doser (Dosatron, Dosmatic,...)<br>☐ Via the drinking water tank<br>☐ This is located at the source<br>☐ This is located at the department	☐ I purchase medicated feed from the compound feed manufacturer<br>☐ Topdressing (powder on top of the feed)<br>☐ Via a doser on the feed line (e.g. KMD-4L from Raijmakers Agro)
31. How do you do the mixing yourself? (For example: I first mix the powder in a bucket of water and then add the water from the bucket to the water tank, I add the powder directly to the water tank, etc.)	……………………………………………………<br>……………………………………………………<br>……………………………………………………<br>……………………………………………………<br>……………………………………………………<br>……………………………………………………<br>……………………………………………………<br>……………………………………………………	……………………………………………………<br>……………………………………………………<br>……………………………………………………<br>……………………………………………………<br>……………………………………………………<br>……………………………………………………<br>……………………………………………………<br>……………………………………………………<br>……………………………………………………
**Question**	**Drinking water**	**Feed**
32. When you add medication to drinking water, do you add anything (e.g. acid or sodium bicarbonate) to make the medication more soluble? If so, what?	……………………………………………………<br>……………………………………………………<br>……………………………………………………<br>……………………………………………………<br>……………………………………………………<br>……………………………………………………<br>……………………………………………………	/
33. When making drinking water/feed medication: do you stir or mix in the pre-solution?	☐ Yes, as long as indicated in the package leaflet<br>☐ Sometimes<br>☐ Never	☐ Yes, as long as indicated in the package leaflet<br>☐ Sometimes

		☐ Never
34. How do you take the amount of medication you need?	☐ With a measuring cup<br>☐ In a bucket<br>☐ In a graduated dosage vessel<br>☐ By a scale<br>☐ I can estimate how much I need by sight	☐ With a measuring cup<br>☐ In a bucket<br>☐ In a graduated dosage vessel<br>☐ By a scale<br>☐ I can estimate how much I need by sight
**Question**	**Drinking water**	**Feed**
35. How often do you usually prepare medicated feed/water? (e.g. 1x per day)	……………………………………………… ……………………………………………… ……………………………………………… ……………………………………………… ……………………………………………… ……………………………………………… ……………………………………………… ……………………………………………… …	……………………………………………… ……………………………………………… ……………………………………………… ……………………………………………… ……………………………………………… ……………………………………………… ……………………………………………… ……………………………………………… ………………………………………………
36. Do you give the medicated water/feed throughout the day, or a few hours a day? (For example: continuous medicated water for 5 days)	☐ All day long<br>☐ Several hours a day	☐ All day long<br>☐ Several hours a day

**Question**	**Drinking water**	**Feed**
37. If you have a metering pump/doser on the waterline/feedline, when was the last time maintenance/calibration was carried out on it?	……………………………………………… ……………………………………………… ……………………………………………… ……………………………………………… ……………………………………………… ……………………………………………… ……………………………………………… ……………………………………………… …	……………………………………………… ……………………………………………… ……………………………………………… ……………………………………………… ……………………………………………… ……………………………………………… ……………………………………………… ……………………………………………… ………………………………………………
38. Do you use gloves when taking water/feed medication?	☐ Always<br>☐ Sometimes<br>☐ Never	☐ Always<br>☐ Sometimes<br>☐ Never

39. Do you use a mask when taking water/feed medication	☐ Always<br>☐ Sometimes<br>☐ Never	☐ Always<br>☐ Sometimes<br>☐ Never

**Question**	**Drinking water**	**Feed**
**Cleaning and disinfection after medication**		
40. Do you clean the utensils used (e.g. the bucket) after taking medication?	☐ Always<br>☐ Sometimes<br>☐ Never	☐ Always<br>☐ Sometimes<br>☐ Never
41. Is drinking water disinfection stopped if you put medication in the drinking water?	☐ Yes<br>☐ No<br>☐ I do not use drinking water disinfection	/
**Own experience with drinking water/feed medication**		
42. Do you sometimes experience problems with the ease of use of feed/drinking water medication? (For example, problems with the workload)	……………………………………………………<br>……………………………………………………<br>……………………………………………………<br>……………………………………………………<br>……………………………………………………<br>……………………………………………………<br>……………………………………………………<br>……………………………………………………	……………………………………………………<br>……………………………………………………<br>……………………………………………………<br>……………………………………………………<br>……………………………………………………<br>……………………………………………………<br>……………………………………………………<br>……………………………………………………
**Question**	**Drinking water**	**Feed**
43. Do you sometimes experience problems with the therapy? (For example, clogging, precipitation, not resolving properly, not tasty, wrong taps opened, ...)	……………………………………………………<br>……………………………………………………<br>……………………………………………………<br>……………………………………………………<br>……………………………………………………<br>……………………………………………………<br>……………………………………………………<br>……………………………………………………	……………………………………………………<br>……………………………………………………<br>……………………………………………………<br>……………………………………………………<br>……………………………………………………<br>……………………………………………………<br>……………………………………………………<br>……………………………………………………<br>……………………………………………………

44. How effective do you find the treatment? (For example, are the pigs always better after the treatment?)	…………………………………………………	…………………………………………………
**Question**	**Drinking water**	**Feed**
45. Do you have a preference for a certain type of drinking water/feed and why?	…………………………………………………	…………………………………………………

Thank you very much for your time! May we contact you later to take feed and/or water samples?

- ☐ **Yes**
- ☐ **No**
